# Predictive potential of dynamic contrast-enhanced MRI and plasma-derived angiogenic factors for response to concurrent chemoradiotherapy in human papillomavirus-negative oropharyngeal cancer

**DOI:** 10.2478/raon-2024-0044

**Published:** 2024-09-15

**Authors:** Alja Longo, Petra Hudler, Primoz Strojan, Gaber Plavc, Lan Umek, Katarina Surlan Popovic

**Affiliations:** Institute of Radiology, University Medical Centre Ljubljana, Ljubljana, Slovenia; Faculty of Medicine, University of Ljubljana, Ljubljana, Slovenia; Institute of Biochemistry and Molecular Genetics, Faculty of Medicine, University of Ljubljana, Ljubljana, Slovenia; Institute of Oncology, Ljubljana, Slovenia; Faculty of Public Administration, University of Ljubljana, Ljubljana, Slovenia

**Keywords:** DCE-MRI, angiogenesis, VEGF, concurrent chemoradiotherapy, response, head and neck squamous cell carcinoma

## Abstract

**Background:**

Dynamic contrast-enhanced magnetic resonance imaging (DCE-MRI) can assess tumour vascularity, which depends on the process of angiogenesis and affects tumour response to treatment. Our study explored the associations between DCE-MRI parameters and the expression of plasma angiogenic factors in human papilloma virus (HPV)-negative oropharyngeal cancer, as well as their predictive value for response to concurrent chemoradiotherapy (cCRT).

**Patients and methods:**

Twenty-five patients with locally advanced HPV-negative oropharyngeal carcinoma were prospectively enrolled in the study. DCE-MRI and blood plasma sampling were conducted before cCRT, after receiving a radiation dose of 20 Gy, and after the completion of cCRT. Perfusion parameters k_trans_, k_ep_, V_e_, initial area under the curve (iAUC) and plasma expression levels of angiogenic factors (vascular endothelial growth factor [VEGF], connective tissue growth factor [CTGF], platelet-derived growth factor [PDGF]-AB, angiogenin [ANG], endostatin [END] and thrombospondin-1 [THBS1]) were measured at each time-point. Patients were stratified into responders and non-responders based on clinical evaluation. Differences and correlations between measures were used to generate prognostic models for response prediction.

**Results:**

Higher perfusion parameter k_trans_ and higher plasma VEGF levels successfully discriminated responders from non-responders across all measured time-points, whereas higher iAUC and higher plasma PDGF-AB levels were also discriminative at selected time points. Using early intra-treatment measurements of k_trans_ and VEGF, a predictive model was created with cut-off values of 0.259 min^−1^ for k_trans_ and 62.5 pg/mL for plasma VEGF.

**Conclusions:**

Early intra-treatment DCE-MRI parameter k_trans_ and plasma VEGF levels may be valuable early predictors of response to cCRT in HPV-negative oropharyngeal cancer.

## Introduction

Head and neck cancers are the seventh most common cancer worldwide, contributing to about 4.5% of global cancer deaths.^1^ The most recent (8th) edition of the Union for International Cancer Control (UICC) staging system distinguishes human papilloma virus (HPV) positive and HPV-negative head and neck squamous cell carcinoma (HNSCC) as distinct entities, with the latter typically associated with poorer prognoses.^2^ In recent decades, non-surgical treatment strategies for locally advanced HPV-negative HNSCC have remained largely unchanged, relying primarily on concurrent chemoradiotherapy (cCRT). The identification of reliable biomarkers for predicting responses to cCRT could aid in stratifying patients for potential individualised treatment adjustments.

Vascular-rich HNSCCs are thought to be more responsive to treatment and show greater sensitivity to radiation compared to less vascular HNSCCs due to enhanced delivery of chemotherapeutic agents and improved oxygenation status.^3^ Vascular characteristics of HNSCC can be investigated using functional MR imaging, specifically dynamic contrast-enhanced MRI (DCE-MRI). In recent years, both pre-treatment and early intratreatment functional MR imaging have shown promising results in predicting cCRT outcomes for HNSCC.^4,5^ Perfusion parameter k_trans_, a measure of capillary permeability, is particularly promising for its potential clinical application in predicting survival and could be used to guide treatment decisions.^5^

Tumour vascularity depends on angiogenesis, a process regulated by various angiogenic factors and cytokines within the tumour microenvironment. Key players in this regulation include vascular endothelial growth factors (VEGFs), which are potent inducers of angiogenesis and vascular permeability. In addition, several other angiogenic factors have been implicated in promoting angiogenesis in HNSCC, including connective tissue growth factor (CTGF), platelet-derived growth factor (PDGF) and angiogenin (ANG), whereas endostatin (END) and thrombospondin-1 (THBS1) have been investigated as endogenous angiogenesis inhibitors.^6,7,8,9^

The objective of this study was to investigate differences in DCE-MRI perfusion parameters and the levels of specific plasma angiogenic factors in HPV-negative oropharyngeal squamous cell carcinoma (OPSCC) patients, distinguishing between responders and non-responders to cCRT. Our goal was to identify measures that could improve patient stratification and enhance the accuracy of outcome prediction.

## Patients and methods

This was a single-centre prospective study conducted at the Institute of Radiology, University Medical Centre Ljubljana, in collaboration with the Institute of Oncology Ljubljana and the Institute of Biochemistry and Molecular Genetics, Faculty of Medicine, University of Ljubljana. The study was approved by the National Medical Ethics Committee of the Republic of Slovenia (No. 0120-247/2019/4, 12 June 2019) and the Committee for Medical Ethics of the Institute of Oncology Ljubljana (OI: 28.5.2019, ERIDEK-0064/2019). Written informed consent was obtained from all patients.

### Patient selection

We consecutively included patients with previously untreated locally advanced HPV-negative OPSCC who were scheduled for curative cCRT. Patients were treated with the intensity-modulated radiotherapy (RT) technique to a radiation dose of 70 Gy in 35 fractions over 7 weeks. Cisplatin 40 mg/m2/week IV or carboplatin 1.5 AUC/week were administered concurrently with RT. HPV status was determined by p16 immunohistochemistry and *in situ* hybridization studies. Patients with concurrent malignancies and those unable to undergo MRI examination were excluded from the study.

### Study design

Patients underwent DCE-MRI before treatment initiation, after receiving 20 Gy of irradiation, and 10–12 weeks after cCRT completion. Peripheral venous blood samples were obtained at each MRI timepoint.

### MR imaging

MRI scans were conducted using a 3.0T MAGNETOM Trio, A Tim System scanner (Siemens Healthineers®, Forchheim, Germany), equipped with a head and neck receive coil. The diagnostic imaging protocol included an axial T2-weighted sequence with short tau inversion recovery (STIR) (TR/TE 5010/71 ms, TI 170 ms, matrix size 256 x 256, slice thickness 3 mm, 10% gap, and field of view (FOV) 18 x 18 cm) and a contrast-enhanced (CE) axial T1-weighted volumetric interpolated breath-hold examination (VIBE) sequence (TR/TE 3.26/1.26 ms, voxel size 1.1 x 0.9 x 1.5 mm, matrix size 288 x 288, and FOV 250 cm^2^). DCE-MRI was performed using a 3D fast low-angle shot (FLASH) sequence optimized for spatial and temporal resolution (TR/TE 5/1.16 ms, matrix size 220 cm^2^, slice thickness 4 mm, temporal resolution 9 s, total acquisition time 6 min). T1 mapping was utilized to convert signal intensities into gadolinium concentration. The T1 map was derived from two pre-contrast flip angle images (6° and 15°). Baseline images were acquired for 27 seconds. Intravenous administration of the paramagnetic contrast medium gadobutrol (Gadovist®, Bayer HealthCare Pharmaceuticals) was performed at a dose of 0.1 mmol/kg body weight with a flow rate of 3.5 mL/s, followed by a 20 mL saline flush. The diagnostic imaging protocol (axial STIR and CE axial T1 VIBE) was employed for pre-treatment tumour evaluation and radiation treatment planning.

### Treatment response

Patients were stratified into two groups based on clinical evaluation. Responders demonstrated complete disappearance of the primary tumour within 10–12 weeks following cCRT. Non-responders encompassed all other patients, including those with partial response, stable disease, or progressive disease.

### DCE-MRI perfusion analysis

We conducted the post-processing of DCE 3D datasets using a commercially available Tissue 4D workflow within the syngo.via medical imaging software (Siemens Healthineers, Forchheim, Germany). We utilized a population-based arterial input function with an intermediate pre-set, and motion correction was automatically conducted using the software algorithm.

Regions of interest (ROI) delineation was verified by two experienced head and neck radiologists (A. L., K. Š. P.). ROI were delineated across VIBE multiple images, resulting in the generation of volumes of interest (VOI). VOI delineation was conducted twice for each examination. Initially, the entire tumour volume was delineated, excluding larger vessels and necrotic regions. These were assessed visually based on their appearance and enhancement characteristics, with large necrotic areas showing little to no enhancement. Subsequently, a smaller tumour volume was delineated, characterized by homogeneous or near-homogeneous enhancement, with a substantial margin from surrounding heterogeneities. The Tofts two-compartment pharmacokinetic model was utilised to derive a set of perfusion parameters, which included the volume transfer constant between blood plasma and extracellular extravascular space (k_trans_), the total extracellular extravascular space (EES) volume fraction (V_e_), the reflux constant (k_ep_) and the initial area under the curve (iAUC) for the selected VOI. The software generated parameter maps depicted as colour-coded images. The mean values of perfusion parameters from the two delineated VOIs were used for analysis.

### Biochemical analysis of plasma angiogenic factors

Blood samples underwent processing to yield 0.5 ml plasma samples, which were immediately stored in the Protein LoBind® Tubes (Eppendorf, Hamburg, Germany) at −80°C. Plasma concentrations of VEGF, CTGF, PDGF-AB, ANG, END, and THBS1 were measured at selected time-points using the enzyme-linked immunosorbent assay (ELISA) technique. The following ELISA Kits were used for the analysis: Human VEGF ELISA Kit (KHG0111), Human PDGF-AB ELISA Kit (EHPDGFAB), Human Angiogenin ELISA Kit (EHANG), Human Endostatin ELISA Kit (EHCOL18A1), Human Thrombospondin 1 ELISA Kit (EHTHBS1), all Invitrogen (Life Technologies Corporation, Carlsbad, CA, USA); and Human CTGF Mini ABTS ELISA Development Kit Catalog# 900-M317 Range (PEPROTech, EC Ltd., London, VB, part of Thermo Fisher Scientific Inc., Waltham, MA, USA).

All steps were performed following the manufacturers' protocols, and each assay was conducted in duplicate or triplicate to ensure accuracy and reliability. To determine the levels of ANG, END, PDGF-AB, and THBS1, the plasma samples underwent dilution at ratios of 8000-, 100-, 2-, and 400-fold using 1x Assay Diluent A or D. The absorbance measurements were conducted using Synergy H4 (Biotek) at 450 or 405 nm with wave-length correction at 650 nm. Standard curves were calculated in GraphPad Prism V. 10 for Windows (GraphPad Software, Boston, MA, USA, www.graphpad.com) using curve fitting analyses, followed by the calculation of results for plasma samples. Sample concentrations were expressed in ng/mL or pg/mL.

### Statistical methods

Shapiro-Wilk tests were used to assess the normality of data distributions. Depending on the results, either Student's t-tests for independent samples or Mann-Whitney U tests were employed to investigate disparities in DCE-MRI perfusion parameters and plasma levels of angiogenic factors between responders and non-responders. P-value below 0.05 was considered significant, while a p-value between 0.05 and 0.10 was regarded as indicative of a trend. Spearman correlation analysis was performed to analyse correlations between DCE-MRI perfusion parameters and biochemical parameters. Predictive models for treatment outcomes were developed using logistic regression analysis and a classification tree model. These models were validated using the leave-one-out strategy. The data analysis was carried out using Python extension libraries, including Orange^10^, Pandas^11^, NumPy^11^, Matplotlib^12^, and Scipy^13^.

## Results

### Clinical characteristics

Twenty-five patients met the inclusion criteria. Initial DCE-MRI was performed on average 11 days (SD 4.2; range 3–18) prior to cCRT initiation, and the intra-treatment DCE-MRI was performed after receiving an average of 21 Gy (SD 2.9; range 18–34) of irradiation. Venous samples were collected an average of 11 days (SD 6.4; range 2–19) prior to cCRT initiation, and the intra-treatment samples were obtained after receiving an average of 23 Gy (SD 5.7; range 18–40) of irradiation. Patient and tumour characteristics are summarised in Table 1.

**Table 1.** Summary of patient, tumour and treatment characteristics

Number of patients	25
Age, median (range)	60 (45–70)
Male gender (%)	25 (100)
Primary location (%)	
Oropharynx	25 (100)
Stage (%)	
III	5 (20)
IVa	8 (32)
IVb	12 (48)
Radiotherapy dose (Gy), median (range)	70 (68–70)
Concomitant chemotherapy, median no. of cycles (range)	6 (1–7)

### Data exclusion and missing values in analysis

Significant discrepancies were noted in the DCE-MRI parameter values for one patient, indicating a possible technical issue. As a result, we decided to exclude this patient with stage III OPSCC, leaving us with 24 patients for analysis. Additionally, there was missing data in the biochemical analysis, with two patients failing to attend the follow-up visit at 10–12-week post-cCRT. Furthermore, some data were missing due to values falling below the detection limit, including 8 samples for ANG and 1 sample for THBS1.

### Treatment outcome

The median follow-up for 24 patients was 30 months (range 17–53 months). At the last follow-up visit, 17 patients were alive, 3 of them with active malignant disease. 7 patients died, with index cancer and infection being the cause of death in 5 and 2 patients, respectively. The latter 2 patients were free of treated cancer at the time of death. Among the participants, 15 were classified as responders (complete response), whereas 9 were categorised as non-responders (incomplete response). All non-responders experienced disease progression during the follow-up period. Of these, one individual was eligible for salvage surgery and remained recurrence-free throughout the follow-up period, while two others developed distant metastases, involving the lung and skin, respectively. Conversely, among the responders, one patient experienced local recurrence and subsequently underwent surgery, while another developed distant metastasis involving the bone and lung. The remaining 13 responders remained disease-free throughout the follow-up period.

### Correlation between perfusion parameters and plasma angiogenic factors

**Figure 1.**
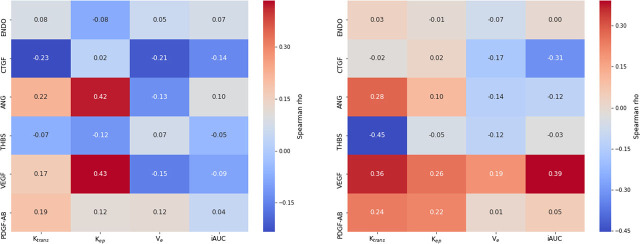
A correlation heatmap visualising perfusion parameters alongside plasma concentrations of angiogenic factors before concurrent chemoradiotherapy (cCRT) initiation (left) and after receiving 20 Gy radiotherapy (right).

Spearman correlation analysis revealed no strong correlations between DCE-MRI perfusion parameters and biochemical parameters. However, moderate positive correlations were observed between k_ep_ and both VEGF and ANG before treatment, as well as between VEGF and both k_trans_ and iAUC after receiving 20 Gy of RT. On the other hand, moderate negative correlation was observed between k_trans_ and THBS1 and also between iAUC and CTGF after receiving 20 Gy of RT. The remaining parameters exhibited weak correlations, including k_trans_ and VEGF before treatment (Spearman rho = 0,166) (Table 2, Figure 1).

**Table 2.** Correlations between dynamic contrast-enhanced magnetic resonance imaging (DCE-MRI) perfusion parameters and biochemical parameters, along with their respective Spearman's rho and p-values

**Time-point**	**DCE-MRI parameter**	**Biochemical parameter**	**Spearman rho**	**p**
**Before treatment**	k_ep_	VEGF	0.433	0.034^*^
	k_ep_	ANG	0.420	0.058
After 20 Gy	k_trans_	THBS1	−0.453	0.026^*^
	k_trans_	VEGF	0.362	0.083
	iAUC	VEGF	0.391	0.056
	iAUC	CTFG	−0.315	0.134

ANG = angiogenin; CTGF = connective tissue growth factor; iAUC = initial area under the curve; THBS1 = thrombospondin-1; VEGF = vascular endothelial growth factor

**Figure 2.**
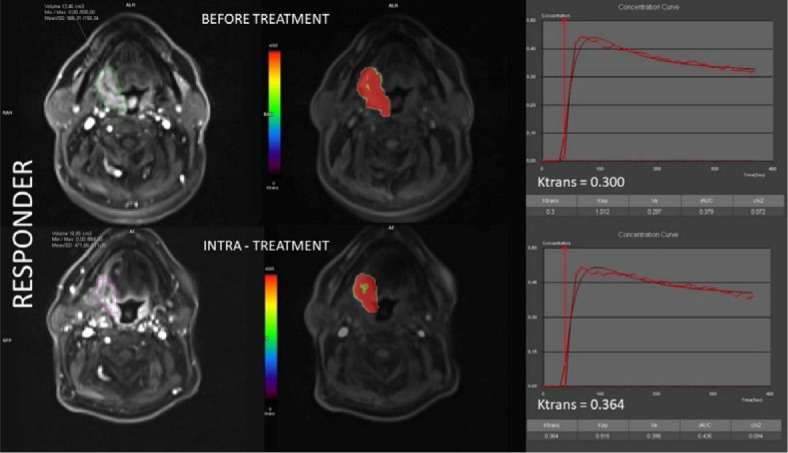
Co-registered volumes of interest (VOI) and color-coded k_trans_ maps, together with concentration curves alongside corresponding k_trans_ values before treatment (upper section) and intra-treatment after receiving 20 Gy radiotherapy (bottom section) in a representative responder patient.

**Figure 3.**
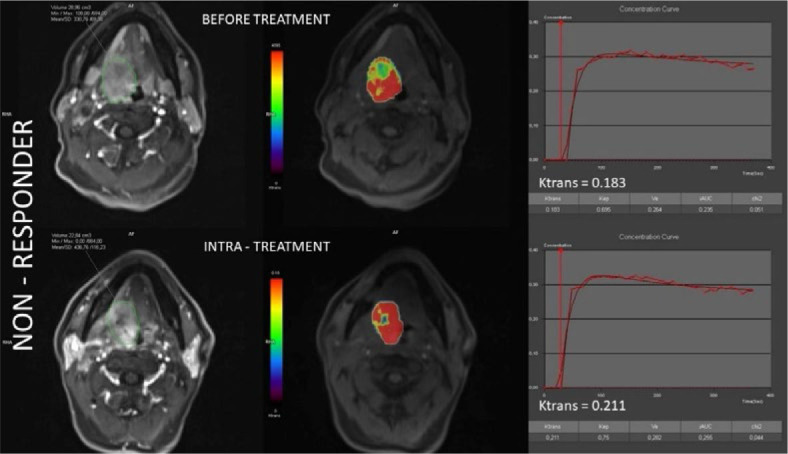
Co-registered volumes of interest (VOI) and color-coded k_trans_ maps, together with concentration curves alongside corresponding k_trans_ values before treatment (upper section) and intra-treatment after receiving 20 Gy radiotherapy (bottom section) in a representative non-responder patient.

Representative co-registered anatomic MR images and colour-coded pharmacokinetic parametric maps of a responder and a non-responder patient before and after 20 Gy RT are shown in Figures 2 and 3, respectively.

### Dynamic contrast-enhanced (DCE) parameters

Compared to non-responders, responders exhibited higher k_trans_ values before treatment (0.270, SD 0.087 min-1 *vs*. 0.169, SD 0.062 min-1, p = 0.006) as well as after receiving 20 Gy of RT (0.289, SD 0.067 min-1 *vs*. 0.215, SD 0.027 min-1, p = 0.007) (Table 3). After receiving 20 Gy of RT, an increase in k_trans_ was observed in both groups. iAUC was larger in responders compared to non-responders before treatment (0.334, SD 0.109 *vs*. 0.220, SD 0.092, p = 0.015) but not after receiving 20 Gy of RT (0.361, SD 0.070 *vs*. 0.336, SD 0.099, p = 0.468). Before treatment, responders exhibited a larger V_e_ compared to non-responders (0.306, SD 0.082 *vs*. 0.237, SD 0.108, p = 0.092); however, this difference was only observed as a trend. After receiving 20 Gy of RT, there was an increase in V_e_ in all patients, but the fractional increase in V_e_ between responders and non-responders was insignificant.

**Table 3.** A summary of tumour perfusion characteristics for responders and nonresponders: mean values, standard deviations (SD), and corresponding p-values

**DCE-MRI parameter**	**Responders (n = 15)**	**Non-responders (n = 9)**	**p**
**k_trans_ (SD)**			
Before treatment	0.270 (0.087)	0.169 (0.062)	0.006^**^
After 20 Gy	0.289 (0.067)	0.215 (0.027)	0.007^**^
Δ	0.019 (0.060)	0.047 (0.078)	0.326
**k_ep_ (SD)**			
Before treatment	0.924 (0.231)	0.817 (0.258)	0.144
After 20 Gy	0.819 (0.229)	0.709 (0.086)	0.065
Δ	−0.105 (0.274)	−0.108 (0.245)	0.980
**V_e_ (SD)**			
Before treatment	0.306 (0.082)	0.237 (0.108)	0.092
After 20 Gy	0.366 (0.086)	0.347 (0.107)	0.636
Δ	0.059 (0.093)	0.109 (0.196)	0.721
**iAUC (SD)**			
Before treatment	0.334 (0.109)	0.220 (0.092)	0.015^*^
After 20 Gy	0.361 (0.070)	0.336 (0.099)	0.468
Δ	0.027 (0.120)	0.116 (0.183)	0.676

iAUC = initial area under the curve

Responders exhibited a trend of higher k_ep_ values compared to non-responders after receiving 20 Gy of RT (0.819, SD 0.229 *vs*. 0.709, SD 0.086, p = 0.065). Before treatment, k_ep_ values did not distinguish between responders and non-responders.

### Biochemical analysis

Plasma levels of VEGF were consistently higher in responders compared to non-responders across all three time points – before treatment (p = 0.011), after receiving 20 Gy of RT (p < 0.001) as well as 10–12 weeks following the completion of cCRT (p = 0.010) (Table 4). There were no significant differences observed in the dynamics of plasma VEGF levels between the two groups (p = 0.462).

**Table 4.** Expression of plasma angiogenic factors for responders and non-responders: mean values, 95% confidence interval (CI) and corresponding p-values

**Plasma Angiogenic Factor**	**Responders (n = 15)**	**Non-responders (n = 9)**	**p**
**VEGF (95% CI) pg/mL**			
Before treatment	66.9 (58.1–75.7)	50.2 (41.8–58.5)	0.011*
After 20 Gy	68.4 (61.0 –75.7)	48.0 (40.4–55.5)	0.0006**
10–12 weeks after cCRT completion	74.7 (50.8–98.6)	49.0 (38.5–59.5)	0.010*
**PDGF-AB (95% CI) pg/mL**			
Before treatment	2826.6 (1909.3–3743.9)	2430.6 (1179.1–3682.1)	0.431
After 20 Gy	2496.0 (2105.8–2886.2)	1799.1 (1319.3–22789)	0.021*
10–12 weeks after cCRT completion	1513.5 (902.7–2124.3)	1468.5 (779.1–2157.9)	0.919
**ANG (95% CI) pg/mL**			
Before treatment	2140.8 (1588.2–2693.4)	1218.0 (223.0 –2213.0)	0.060
After 20 Gy	2124.1 (1653.4–2594.9)	1438.0 (405.6–2470.4)	0.131
10–12 weeks after cCRT completion	1898.1 (1248.7–2547.6)	1384.8 (255.7–2513.9)	0.347
**THBS1 (95% CI) ng/mL**			
Before treatment	1113.2 (1069.3–1157.2)	1115.6 (1055.8–1175.5)	0.943
After 20 Gy	1112.3 (1051.5–1173.1)	1162.7 (1064.2–1261.2)	0.257
10–12 weeks after cCRT completion	1061.2 (1008.8–1113.8)	1136.8 (1002.9–1270.7)	0.210
**END (95% CI) pg/mL**			
Before treatment	64792.6 (52731.7–76853.4)	59350.6 (47299.1–71402.1)	0.521
After 20 Gy	71269.2 (55039.7–87498.7)	59636.7 (48124.0 –71149.5)	0.283
10–12 weeks after cCRT completion	84744.3 (65061.5–104427.2)	74754.7 (54514.5–94994.9)	0.475
**CTGF (95% CI) pg/mL**			
Before treatment	32.6 (13.9–51.2)	24.4 (14.4–34.5)	0.976
After 20 Gy	31.2 (24.1–122.8)	23.5 (12.3–34.8)	0.721
10–12 weeks after cCRT completion	26.1 (15.7–36.6)	23.2 (15.1–31.3)	1

ANG = angiogenin; cCRT = concurrent chemoradiotherapy; CTGF = connective tissue growth factor; END = endostatin; PDGF-AB = platelet-derived growth factor AB; THBS1 = thrombospondin-1; VEGF = vascular endothelial growth factor

Plasma levels of PDGF-AB showed a decreasing trend across the three time points for all patients, with no significant difference observed in the dynamics between responders and non-responders. After receiving 20 Gy of RT, plasma levels of PDGF-AB were higher in responders compared to non-responders (p = 0.021), whereas no significant difference was observed between the two groups before treatment (p = 0.431) and 10–12 weeks after CRT completion (p = 0.919).

Apart from several values falling below the detection limit, there was considerable variation in the measured levels of plasma ANG among all patients. No notable dynamics or significant differences were observed between responders and non-responders. Plasma levels of THBS1 remained stable across the three time points, with no discernible differences observed between responders and non-responders. An insignificant increase in plasma levels of END was observed across the three time points in all patients, with no noticeable differences between responders and non-responders. Plasma levels of CTGF displayed considerable variability, with no notable differences observed between responders and non-responders (Table 4).

### Predicting treatment response

Based on Spearman correlation analysis and descriptive statistics for DCE-MRI and biochemical parameters between responders and non-responders, we selected two variables for inclusion in statistical modelling to predict treatment outcomes, namely k_trans_ and VEGF as measured after receiving 20 Gy of RT. The multivariate logistic regression (LR) analysis, which incorporated two predictor variables, k_trans_ and plasma VEGF after receiving 20 Gy of RT, yielded a classification accuracy (CA) of 0.708 and area under the ROC curve (AUC) of 0.859. Notably, the variable VEGF achieved statistical significance (p = 0.033), whereas the variable k_trans_ approached significance (p = 0.056).

A univariate LR analysis utilizing only the variable k_trans_, measured after receiving 20 Gy of RT, exhibited a higher CA of 0.792, but a significantly lower AUC of 0.625. Notably, the variable k_trans_ demonstrated statistical significance (p = 0.019).

**Figure 4.**
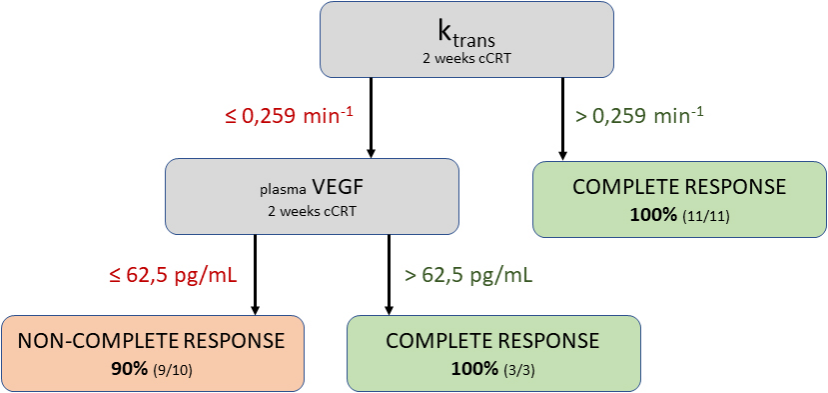
Graphical presentation of the classification tree model. cCRT = concurrent chemoradiotherapy; VEGF = vascular endothelial growth factor

The classification tree model, using the same two variables, achieved CA of 0.833 and AUC of 0.881. The classification tree (Figure 4) structure consists of one terminal node on the first branch and two terminal nodes on the second. Variable k_trans_ was found to be the most influential in determining treatment response with a cut off value of 0.259 min^−1^, followed by variable VEGF with a cut off value of 62.5 pg/mL.

## Discussion

In the present study we confirmed the efficacy of DCE-MRI in combination with plasma angiogenic factor analysis before and early during cCRT in predicting response to cCRT in patients with HPV-negative OPSCC.

The key DCE-MRI perfusion parameter k_trans_ reflects microvascular permeability and blood flow within the primary tumour.^10^ In our study, we showed that responders to cCRT had significantly higher pre-treatment k_trans_ values compared to non-responders. This finding is in line with several previous reports on HNSCC and other malignancies that linked higher pre-treatment k_trans_ values to longer survival and better local control.^10,12,13,14,15,16^ This supports the premise that higher perfusion within tumours is associated with increased oxygenation levels, which in turn enhances radiosensitivity and improves chemotherapeutic drug delivery.^3^ A previous study exploring intra-treatment dynamics of DCE-MRI parameters in HNSCC has shown a larger fractional increase in primary tumour k_trans_ and V_e_ in responders versus non-responders.^17^ Similarly, patients achieving locoregional control demonstrated persistently elevated or increasing levels of intra-treatment blood flow (BF), volume (BV), and permeability surface (PS), whereas one study reported a decrease in k_trans_ after 2 weeks of cCRT, which was linked to significantly reduced survival.^4,15^ Interestingly, we observed an increase in k_trans_ values among both responders and non-responders after receiving 20 Gy RT. This observation aligns with the concept that low-dose radiation induces proangiogenic signalling, promotes angiogenesis, improves tumour perfusion, enhances vascular permeability, reduces hypoxia, and subsequently leads to vascular normalisation.^18^ Our results suggest that this effect exists independently of the final treatment outcome. iAUC is influenced by permeability, blood flow, and washout, providing an alternative parameter to k_trans_ in clinical trials.^19,20^ Our study showed that pre-treatment iAUC was higher in responders compared to non-responders, mirroring the k_trans_ results. However, unlike k_trans_, this distinction disappeared after receiving 20 Gy of RT.

In addition to the increase in blood flow and vascular permeability, radiation-induced cellular degradation leads to the expansion of interstitial space.^17^ DCE-MRI parameter V_e_ reflects the EES, which consists of interstitial fluid and connective tissue and differs significantly in neoplastic tissues from most normal tissues.^21^ Earlier research has indicated a mostly positive correlation between elevated pre-treatment V_e_ and favourable outcomes, but these findings were inconsistent.^12,13,14^ One study reported a greater fractional increase in V_e_ among responders compared to non-responders.^17^ Similarly, in the present study we observed a tendency for a larger pre-treatment V_e_ in responders compared to non-responders. We observed a rise in V_e_ across all patients after receiving 20 Gy of RT. However, both the fractional increase of V_e_ and the absolute difference in V_e_ values after receiving 20 Gy of RT between responders and non-responders were found to be insignificant. We assume that the differences in the EES of tumours that may exist before treatment between responders and non-responders are diminished by the shared effects of radiotherapy.

VEGF is a main driver of angiogenesis, known to be upregulated in the plasma of various cancer patients.^22,23,24^ Several studies have investigated the relationship between VEGF expression in tissue specimens and DCE-MRI parameters, especially k_trans_, resulting in conflicting findings.^25,26,27,28^ VEGF has been investigated as a potential biomarker in various cancer types, including HNSCC.^29,30^ High VEGF expression levels in serum have been linked to increased tumour growth, invasiveness, and a higher risk of metastasis.^31^ To our knowledge, no studies to date have explored the combination of DCE-MRI with plasma angiogenic factors as potential prognostic biomarkers. According to literature, the average reported healthy plasma VEGF value is 42 pg/mL (SD 20, range 9–126 pg/mL), whereas in cancer it is 74 pg/mL (SD 116, range 19–730 pg/mL), the latter displaying considerable variability.^23^ A pre-therapeutic plasma VEGF threshold below 26 ng/L was reported to correlate with a more favourable outcome in HNSCC.^30^ Our results, on the other hand, indicate a different trend - plasma VEGF levels were consistently higher in responders when compared to non-responders across all three time points – before treatment, after receiving 20 Gy of RT, as well as 10–12 weeks following the completion of cCRT. Explaining the rationale behind this observation is challenging, as the factors affecting plasma VEGF levels are highly multifactorial. Compared to the total amount of VEGF in the human body, tumour contributes to a relatively small percentage of VEGF. Besides the tumour, skeletal muscles seem to serve as a significant reservoir for VEGF in the body. Moreover, the renal clearance of VEGF should be considered as a contributing factor to plasma VEGF levels.^23^ Although it may be overly ambitious to assume a direct relationship between tumour blood flow, permeability and circulating plasma VEGF levels, the observed higher plasma VEGF levels in responders merit consideration and further investigation. Since lower-doses of radiation induce proangiogenic signalling and promote angiogenesis, elevated levels of VEGF, as a crucial player in angiogenesis, may manifest in the circulating plasma as a reflection of this process.^18^ This could explain why the correlation between VEGF and k_trans_/iAUC appears only after the start of the treatment. It could also provide insight into why these two parameters performed the best when conducting statistical modelling.

PDGF promotes tumour-associated angiogenesis by up-regulating VEGF production.^31,32^ A sole notable distinction between responders and non-responders emerged at the intra-treatment time-point for plasma PDGF-AB, which displayed higher levels among responders. This observation emerged in the same intra-treatment time point where elevated plasma VEGF levels appeared as effective predictors of complete response. This further suggests that physiological processes occurring early during cCRT may be reflected in the plasma levels of these two pro-angiogenic factors. Apart from VEGF and PDGF-AB, none of the analysed angiogenic factors exhibited significant promise for predicting response.

The most effective multivariate predictive models incorporated intra-treatment plasma VEGF and k_trans_ values. Both logistic regression and classification tree models outperformed the univariate models that used only the k_trans_ variable, indicating that plasma VEGF levels provide significant additional predictive value in determining response. Based on our analysis, the classification tree model utilising intra-treatment plasma VEGF and k_trans_ emerged as the best-performing model.

While this study has several limitations, notably its small sample size and the lack of healthy controls for angiogenic factors, our study cohort stands out for its high homogeneity in tumour stage and HPV status, making it unique compared to similar studies. Validating our findings would necessitate a larger study, including healthy controls. Additionally, it is important to note that absolute values of perfusion parameters can vary depending on the post-processing software used for pharmacokinetic modelling in DCE-MRI. Therefore, caution is needed when comparing these values across different studies.

In conclusion, the early intra-treatment DCE-MRI parameter k_trans_ and plasma VEGF levels emerged as the two most robust predictors for treatment response to cCRT in HPV-negative oropharyngeal cancer, yielding effective predictive models with cut-off values of 0.259 min^−1^ for k_trans_ and 62.5 pg/mL for plasma VEGF.
